# Outbreak of Rabbit Hemorrhagic Disease Virus 2 Infections, Ghana

**DOI:** 10.3201/eid2707.210005

**Published:** 2021-07

**Authors:** Aruna Ambagala, Patrick Ababio, Lindsey Lamboo, Melissa Goolia, Oliver Lung, Yohannes Berhane, Theophilus Odoom

**Affiliations:** National Centre for Foreign Animal Disease, Canadian Food Inspection Agency, Winnipeg, Manitoba, Canada (A. Ambagala, L. Lamboo, M. Goolia, O. Lung, Y. Berhane);; Veterinary Services Directorate, Accra Laboratory, Accra, Ghana (P. Ababio, T. Odoom)

**Keywords:** Canada, Ghana, hemorrhagic diseases, Lagovirus, outbreaks, rabbit hemorrhagic disease, rabbit hemorrhagic disease virus 2, rabbits, RHDV2, veterinary diseases, viruses

## Abstract

In September 2019, high mortality in commercial rabbits was reported in the Greater Accra Region of Ghana. Rabbit hemorrhagic disease virus 2 phylogenetically related to isolates from 2015–2017 outbreaks in the Netherlands was confirmed as the causative agent. The virus has not yet been detected in native rabbits in Ghana.

Rabbit hemorrhagic disease (RHD) is an acute, fatal, highly contagious viral hepatitis in European rabbits (*Oryctolagus cuniculus*) (*1*). It causes severe economic losses in the rabbit meat and fur industries and can have a substantial negative ecological impact on wild rabbit populations and their predators. The causative agent, RHD virus (RHDV; family *Caliciviridae*, genus *Lagovirus*) has a single-stranded positive-sense RNA genome ≈7.4 kb in length. Pathogenic RHDV strains exist in 2 main genotypes, GI.1 and GI.2 (RHDV2). RHDV GI.1 has several variants: GI.1a (proposed as G6/RHDVa), GI.1b (G1), GI.1c (G2), and GI.1d (G3–G5) (*2*). It is considered enzootic in domestic and wild European rabbits in Asia and Europe; sporadic outbreaks occur in the Americas, Middle East, and Africa (*1*). RHDV2 GI.2, a variant first reported in France in 2010 (*3*), differs antigenically from RHDV GI.1 and is therefore considered a distinct serotype. RHDV2, which has replaced RHDV GI.1 in many countries in Europe, infects rabbits of all ages and crosses the species barrier to affect non-European rabbit species (*4*).

Rabbit production, because of its low costs, has been promoted to reduce poverty in Africa but has been threatened by RHD outbreaks since the late 1980s. In 2015, RHDV2 was detected on Tenerife in the Canary Islands (*5*). In April 2017, an outbreak was reported in northern Morocco (*6*) caused by a recombinant GI.1b/GI.1b/GI.2 RHDV2 strain closely related to isolates identified in Portugal in 2014. During 2015–2018, RHDV2 strains similar to those circulating in France, Portugal, Spain, and Finland were isolated in field rabbits in Tunisia (*7*). Recently, rabbit farms in West Africa have been severely affected by RHD. Using competitive ELISA, researchers in Benin in July 2015 confirmed an RHD outbreak in the coastal city Cotonou (*8*). In February 2016, several RHD outbreaks detected by using real-time reverse transcription PCR (rRT-PCR) in Korhogo in northern Côte d’Ivoire, were resolved but the virus and rabbit species were not well characterized (*8*). On June 3, 2020, Senegal reported its first RHDV2 outbreak, which killed many farmed Flemish giant, checkered giant, and Fauve de Bourgogne rabbits (*8*). Nigeria’s first reported outbreak started in September 2020 at rabbit farms in Kwara and Oya, southwestern states bordering Benin (*8*).

A September 2019 RHDV2 outbreak sickened 11,350 rabbits and killed ≈6,000 in commercial rabbit farms in Dansoman, Korle-Bu, Mamprobi, and Banana Inn in the Ablekuma Central District, Greater Accra Region, Ghana. Clinical signs included dullness, anorexia, convulsion, dyspnea, bloody nasal discharge, mucus in feces, and paralysis. Postmortem examinations revealed multifocal petechial hemorrhages in multiple organs, congested and edematous lungs, pulmonary edema with tracheal foam, splenomegaly, and pale livers with areas of necrosis. Tissue samples from affected animals submitted to the Accra Veterinary Laboratory tested positive for RHDV2. The disease quickly spread to other areas of Ghana, including the Ashanti Central Eastern, Bono, and Ahafo regions. We sent 35 rRT-PCR–positive liver, lung, and spleen samples from different regions of Ghana (Appendix Figure 1) to the National Centre for Foreign Animal Disease in Winnipeg, Manitoba, Canada, where rRT-PCR testing confirmed RHDV2 (*9*).

We extracted nucleic acid from 1 of the samples (RHDV2 Ghana-15 2019; Dansoman, Ghana) with a cycle threshold of 12.73 and subjected it to whole-genome sequencing on an Illumina MiSeq instrument (https://www.illumina.com) after targeted cDNA conversion as described elsewhere (*10*) with modifications. The whole genome sequence (GenBank accession no. MW118115) closely resembled (98.84%) that of RHDV2 isolate RHDV-NL2016 (accession no. MN061492) from the 2015–2017 outbreak in the Netherlands (Appendix Figure 2). We later subjected 14 additional independent samples from different locations in the greater Accra region to whole-genome sequencing (accession no. MW789232–245) and found they were ≥99.73% identical at the nucleotide level to RHDV2 Ghana-15 2019, suggesting that this outbreak was caused by a single incursion.

The viral protein 60 gene of RHDV2 Ghana-15 2019 showed 98.73% identity to the same gene in the isolate from the Netherlands (accession no. MN061492), 96.32% to the isolate (accession no. MF407653) from Spain, and only 94.70% to the isolate from Tunisia (accession no. MK629981; Figure). No sequence information was available from the previous RHDV2 outbreaks in West Africa, and therefore it is not possible to comment on the relatedness of the recent RHDV2 outbreaks in this region. Introduction of RHDV2 at the port cities in West Africa suggests that the virus likely entered each country through imported rabbits or rabbit byproducts and was not spread by rabbits moving across land borders. The outbreak in Ghana continues, and the Ghana Veterinary Authority is considering vaccination to protect local rabbit populations. So far, no RHD cases have been reported in native rabbits in Ghana, but the lack of infections could be simply because native rabbits have not yet been exposed to the virus, which could change in the future.

**Figure:**
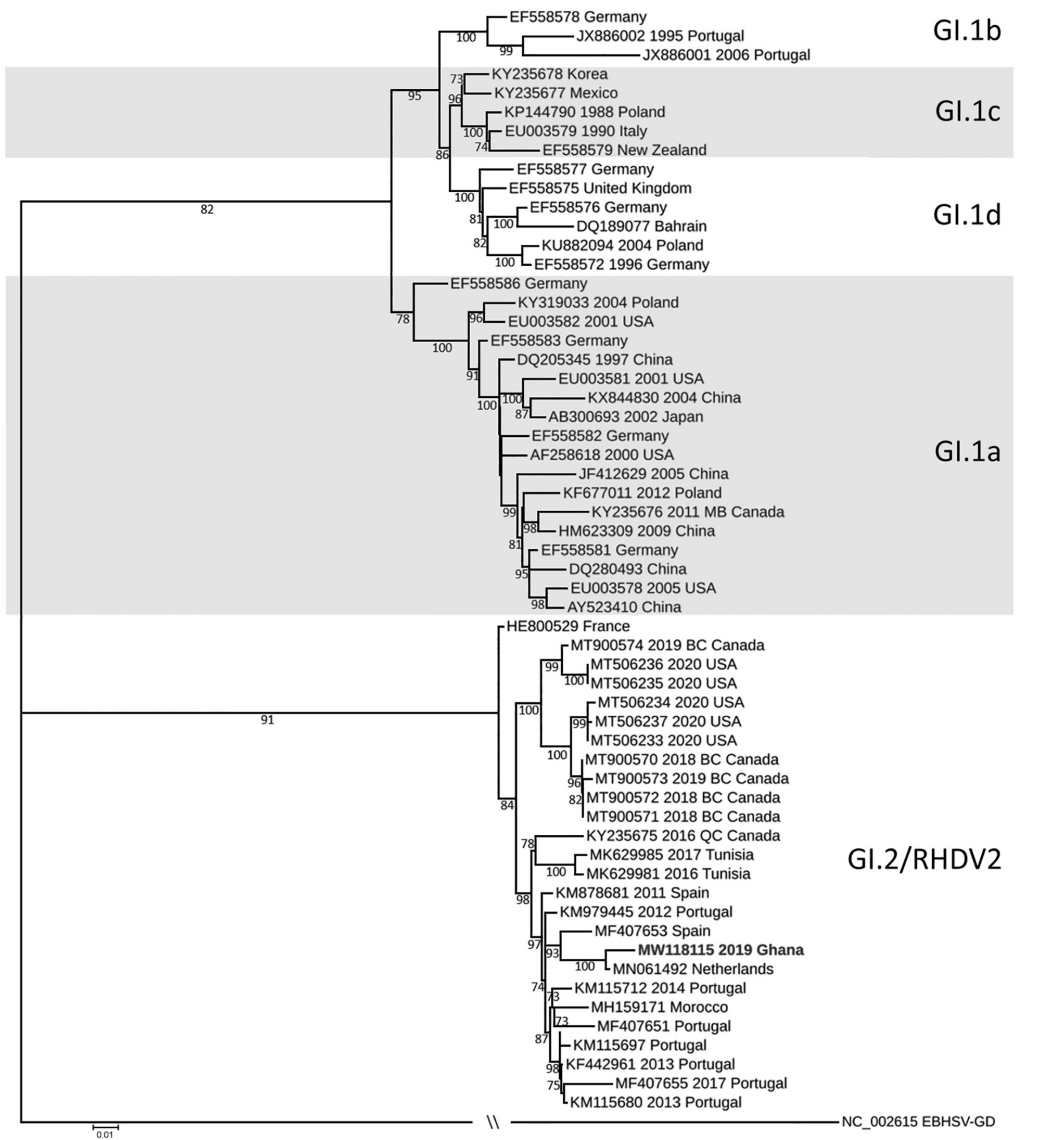
Maximum-likelihood phylogenetic tree of the viral protein 60 region of rabbit hemorrhagic disease virus from Ghana (boldface) and reference sequences. We downloaded sequences directly from or extracted them from the whole genome sequences downloaded from GenBank. We aligned sequences in Geneious Prime (Geneious, https://www.geneious.com) and constructed the phylogenetic tree with the IQ-TREE Web server (http://iqtree.cibiv.univie.ac.at), using 1,000 bootstrap replicates as indicated in the tree. We then visualized the phylogenetic tree using iTOL (https://itol.embl.de). Scale bar indicates branch length.

Appendix Additional geographic and genomic information about the 2019 rabbit hemorrhagic disease virus outbreaks in Ghana.
