# Supplementary material for: Outbreak of Rabbit Hemorrhagic Disease Virus 2 Infections, Ghana
Source: Emerg Infect Dis. 2021 Jul;27(7):1999–2002. doi: 10.3201/eid2707.210005 (PMC8237895; doi:10.3201/eid2707.210005)
Supplement: Appendix — Additional geographic and genomic information about the 2019 rabbit hemorrhagic disease virus outbreaks in Ghana. [file 21-0005-Techapp-s1.pdf]

Article DOI: https://doi.org/10.3201/eid2707.210005

Outbreak of Rabbit Hemorrhagic Disease Virus 2, Ghana

Appendix.

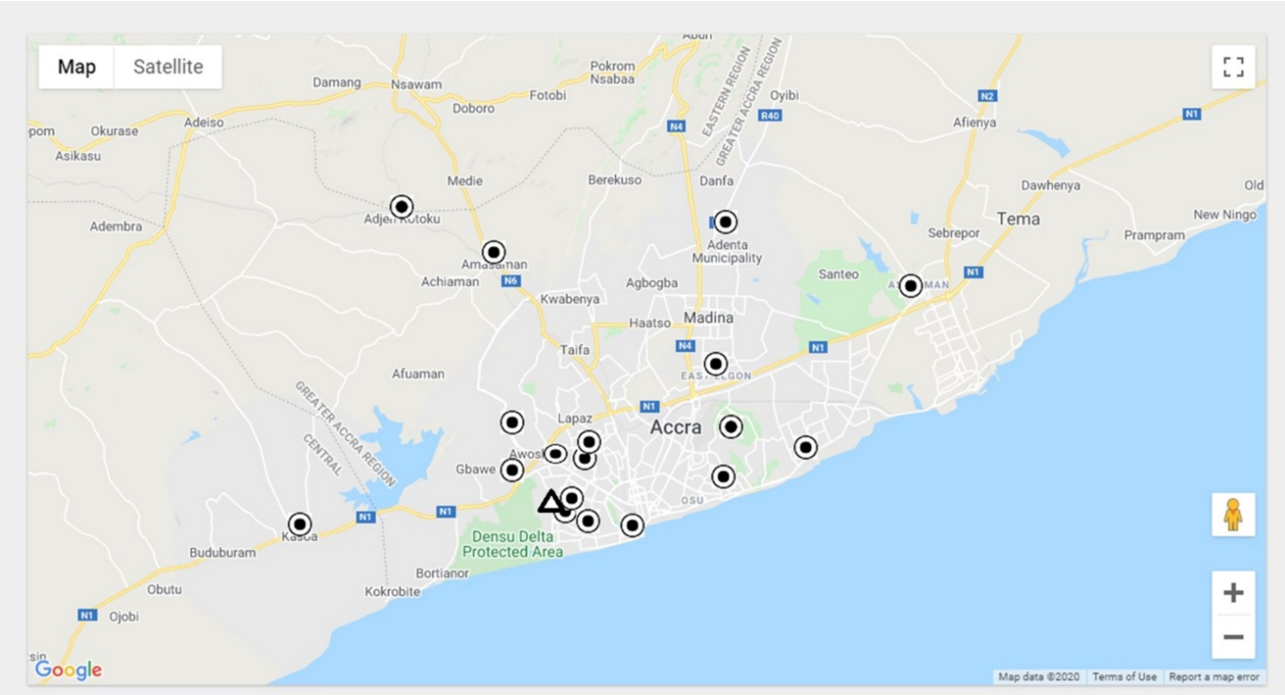

Appendix Figure 1. Locations of rabbit hemorrhagic disease virus outbreaks (dots) in September 2019 in Accra, Ghana. We collected 35 samples from these locations and analyzed them using real-time reverse transcription PCR assays specific for rabbit hemorrhagic disease virus 2. The location of the sample subjected for whole genome sequencing for the first time is marked with a triangle. The satellite figure map was produced from Google Maps (2020 map data; https://www.google.com/maps/place/Accra,+Ghana) in compliance with the terms of service as outlined at https://www.google.ca/permissions/geoguidelines.html with modifications.

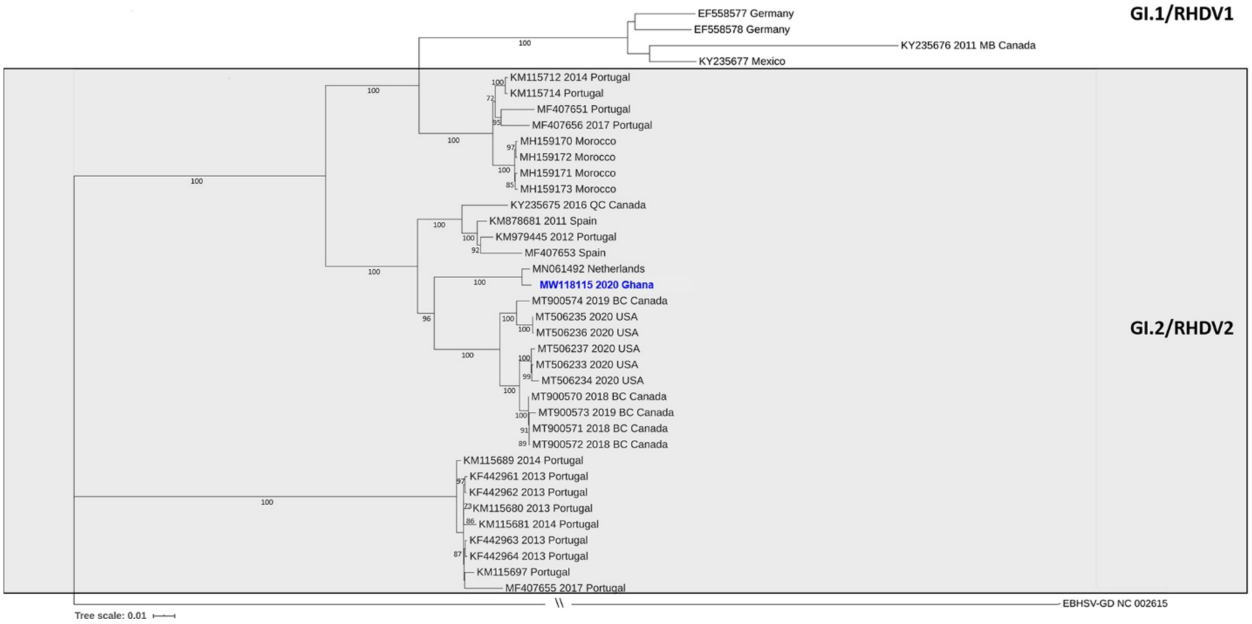

Appendix Figure 2. Maximum-likelihood phylogenetic tree of the whole genome sequences of various isolates of rabbit hemorrhagic disease virus. We downloaded whole genome sequences from GenBank and aligned them in Geneious Prime version 2020.1.1 (Geneious; https://www.geneious.com), then constructed the phylogenetic tree using the IQ-TREE Web server (http://iqtree.cibiv.univie.ac.at), using the SYM+I+G4 nt substitution model and 1000 bootstrap replicates as indicated in the tree. We then visualized the phylogenetic tree using iTOL (http://iqtree.cibiv.univie.ac.at). Ghana isolate is shown in blue font. Genotypes are indicated on the right. For clarity, only a limited number of rabbit hemorrhagic disease virus GI.1 whole genome sequences are depicted.
